# What Are the Trends in Tonsillectomy Techniques in Wales? A Prospective Observational Study of 19,195 Tonsillectomies over a 10-Year Period

**DOI:** 10.1155/2015/747403

**Published:** 2015-11-26

**Authors:** Hussein Walijee, Ali Al-Hussaini, Andrew Harris, David Owens

**Affiliations:** Department of Otorhinolaryngology, Head and Neck Surgery, University Hospital of Wales, Cardiff CF14 4XW, UK

## Abstract

There are a multitude of techniques to undertake tonsillectomy, with hot techniques such as diathermy and coblation being associated with a higher risk of secondary haemorrhage. The UK National Prospective Tonsillectomy Audit (2004) advocated cold steel dissection and ties to be the gold standard. This prospective observational study investigates the trends in tonsillectomy techniques across Wales in the last decade to establish if surgeons have adhered to this national guidance. Data relating to tonsillectomy were extracted over a 10-year period from 1 January 2003 to 31 December 2012 from the Wales Surgical Instrument Surveillance Programme database. A total of 19,195 patients were included. Time-series analysis using linear regression showed there was an increase in the number of bipolar diathermy tonsillectomies by 84% (Pearson's *r* = 0.762, *p* = 0.010) and coblation tonsillectomies by 120% (*r* = 0.825, *p* = 0.003). In contrast, there was a fall in the number of cold steel dissection tonsillectomies with ties by 60% (*r* = −0.939, *p* < 0.001). This observational study suggests that the use of bipolar and coblation techniques for tonsillectomy has increased. This deviation from national guidance may be due to these techniques being faster with less intraoperative bleeding. Further study for the underlying reasons for the increase in these techniques is warranted.

## 1. Introduction

Tonsillectomy is one of the oldest [1] and commonest [2, 3] surgical procedures performed by otorhinolaryngologists worldwide. The main indications for this procedure comprise recurrent tonsillitis and upper airway obstruction including obstructive sleep apnoea. Over time various techniques have been developed and employed in an attempt to reduce haemorrhage rates and improve postoperative morbidity, in particular postoperative pain.

Tonsillectomy can be performed in many different ways depending on the preference and experience of the surgeon. Generally, it may be divided into two stages: excision of the tonsil followed by control of bleeding. However, newer techniques combine these stages so that they are undertaken simultaneously. Cold dissection tonsillectomy involves cutting the pharyngeal mucosa with scissors followed by blunt dissection of the tonsil from the lateral pharyngeal wall, employing no form of heat or cautery. Haemostasis can then be achieved by ligatures or sutures [4].

Diathermy tonsillectomy is a technique that utilises electric current to cut tissue and coagulate blood vessels. Thus, the tonsil is excised and haemostasis is secured simultaneously. This has been advocated to reduce operative time [5]. Coblation is a technique that involves passing radiofrequency energy through a conductive medium such as isotonic sodium chloride creating a plasma field. This results in energetic charge carrying ions that have sufficient energy to break organic molecular bonds, providing simultaneous dissection and coagulation [6] at a temperature of 60–70°C. This is in contrast to monopolar cautery, which generates temperatures of up to 400–600°C. The lower operating temperatures are thought to reduce thermal damage to adjacent tissues and therefore cause less pain postoperatively and improve healing [7].

Although tonsillectomy is a worthwhile surgical intervention when indicated [8], it is not without its complications. The most serious risk associated with the procedure is postoperative haemorrhage, and a multitude of reports have discussed its relationship to operative technique [9, 10]. Different techniques have attracted controversy around their complication rates, in particular the incidence of haemorrhage. This controversy perseveres due to the difficulty in reliably proving or disproving a small difference in the rate of a relatively uncommon complication [11].

Notably, the UK National Prospective Tonsillectomy Audit [9] included 33,921 patients that underwent tonsillectomy between July 2003 and September 2004. This study found an overall haemorrhage rate around three times higher with “hot” surgical techniques for both dissection and haemostasis (diathermy or coblation) than with cold steel tonsillectomy without any use of diathermy. When cold steel was used for dissection and bipolar diathermy only for haemostasis the relative risk of postoperative haemorrhage was around 1.5. The study, however, reported no strong statistical evidence for variations in the risk of return to theatre among most techniques. The authors concluded by recommending that bipolar diathermy and coblation methods should be used with appropriate caution and only after proper training. This is in accordance with several large cohort studies published in the last decade, which also demonstrate an increased risk for the occurrence and/or severity of posttonsillectomy haemorrhage when hot instruments are utilised [12, 13].

Cold steel dissection with cold haemostasis is considered the gold standard technique [14]. However, there is an increasing pressure to reduce costs, operating time, hospital stay, and postoperative morbidity. This prospective observational study aims to investigate the trends in tonsillectomy techniques utilised across Wales in the last decade to explore if otorhinolaryngologists have adhered to or deviated from national guidance favouring cold steel dissection and cold haemostasis above hot tonsillectomy techniques.

## 2. Materials and Methods

Data were obtained from the Surgical Instrument Surveillance Programme (SISP) database [15] and the Patient Episode Database for Wales (PEDW) [16]. The SISP was established in 2003 to monitor the use of single-use instruments in all tonsil and adenoid surgery in Wales across 18 hospitals. Single-use instruments were introduced in Wales following fear of Creutzfeldt-Jakob prion transmission via reusable instruments. The SISP was designed to monitor the primary outcomes of instrument failure and postoperative haemorrhage. The data is anonymised and coded, and for this reason ethical approval was not required for the reuse of anonymous and open access population data.

Data relating to tonsillectomy were extracted using the Office of Population, Censuses and Surveys Classification of Surgical Operations and Procedures 4th revision (OPCS-4) [17] identifier code F34. Data were extracted over a 10-year period from 1st January 2003 to 31st December 2012 from the SISP database. Hospital stay data for tonsillectomy were extracted from the PEDW for the period between 2003 and 2012. Patients who had a tonsillectomy for recurrent infection and airway obstruction were included in the study. Patients undergoing an adenoidectomy alone, tonsillectomy as part of diagnosis or treatment of cancer, or tonsillectomy as part of palatal surgery and incomplete datasets were excluded. There were no recorded cases of tonsillotomy/intracapsular tonsillectomy.

The respective number of patients was expressed as a percentage of the total number of tonsillectomies performed using each technique every individual year. Time-series analysis with a linear regression model was used to detect a change in the percentage of tonsillectomies performed using the technique in question. Pearson correlation analysis and the independent sample *t*-test were used to determine significance. Data were analysed in Microsoft Excel 2013 and IBM SPSS Statistics 20. A *p* value of <0.05 was considered statistically significant.

## 3. Results and Discussion

A total of 25,592 patients were identified from the SISP database as having undergone a tonsillectomy between 1st January 2003 and 31st December 2012; 6,397 patients were excluded from the study. Amongst those excluded, 5,676 patients had actually undergone an adenoidectomy as the only procedure, a tonsillar biopsy, and tonsillectomy for known cancer, uvulopalatoplasty, or poorly documented indication; the remaining 721 patients were excluded for the lack of documentation of the technique employed during the procedure. A total of 19,195 patients who underwent a tonsillectomy for recurrent tonsillitis (91%), peritonsillar abscess (5%), and upper airway obstruction (4%) were included in this study. Overall, gender variation within the cohort showed a significant female predominance with a gender ratio of 1.87 : 1 (*p* < 0.001). The median age of patients included in this study was 16 years (range: 2–74 years).

**Figure 1:**
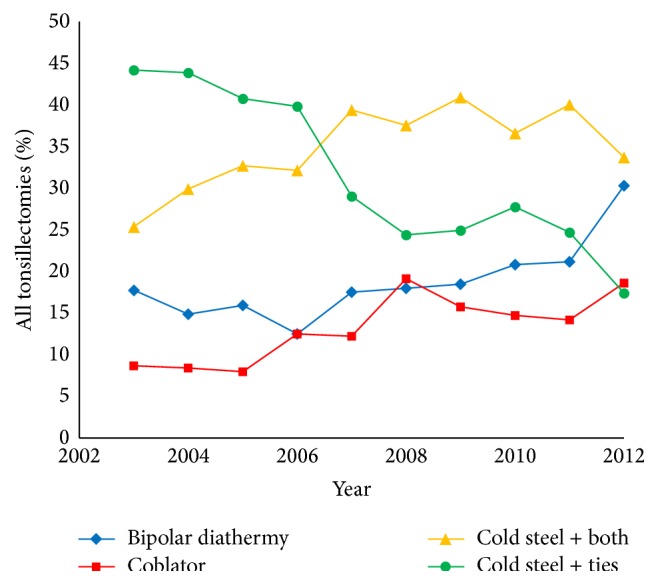
Time-series analysis of tonsillectomy techniques utilised in Wales between 2003 and 2012.

Time-series analysis using a linear regression model demonstrated that, between 2003 and 2012, there was a significant increase in the number of coblation tonsillectomies by 120% (Pearson's *r* = 0.825, *p* = 0.003) from an initial 8.3% in 2003 to 18.3% of all tonsillectomies in 2012. Furthermore, there was a significant increase in the use of bipolar diathermy by 84% (*r* = 0.762, *p* = 0.010). In contrast, time-series analysis demonstrated a significant fall in the number of cold steel tonsillectomies with ties by 60% (*r* = −0.939, *p* < 0.001) from an initial 45% in 2003 to 18% of all tonsillectomies in 2012. Cold steel dissection with the use of both diathermy and ties for haemostasis was the most prevalent technique in 2012, followed by bipolar diathermy. Additionally, there was a significant decrease in the use of laser, ultrasonic, and monopolar diathermy tonsillectomy techniques.  Table 1 summarises the trends of tonsillectomy techniques utilised between 2003 and 2012 in Wales.  Figure 1 depicts the trends in the techniques of coblation, bipolar diathermy dissection and haemostasis, cold steel dissection with ties, and cold steel dissection with haemostasis with ties and bipolar diathermy, in Wales between 2003 and 2012.

Time-series analysis using a linear regression model was also undertaken to examine the trends, if any, in tonsillectomy techniques utilised by otolaryngology consultants and specialist registrars (trainees) within the ten-year study period. With respect to the use of coblation, there was no significant change of its frequency of use by consultants but a significant increase of its use by specialist registrars (*r* = 0.846, *p* = 0.002). Although there was a large increase in the use of coblation by specialist registrars within the study period, this reflected virtually no use of this technique by this group in 2003 and it comprising fewer than 10% of all tonsillectomies performed by specialist registrars in 2012.

**Figure 2:**
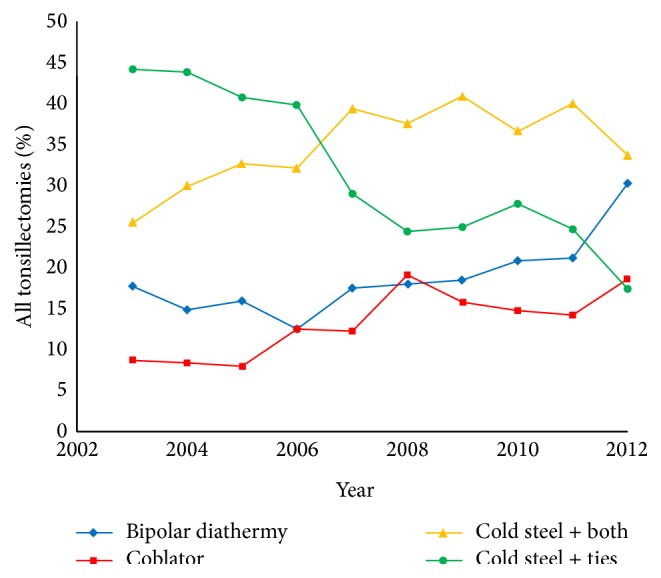
Time-series analysis of tonsillectomy techniques utilised by consultants in Wales between 2003 and 2012.

**Figure 3:**
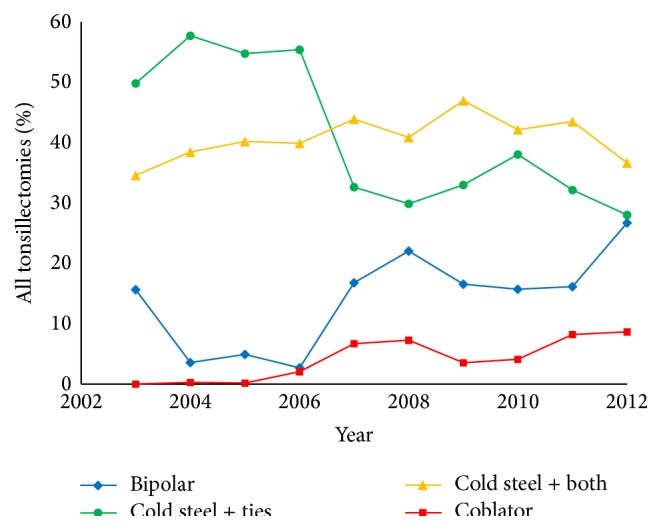
Time-series analysis of tonsillectomy techniques utilised by specialist trainees in Wales between 2003 and 2012.

Time-series analysis also elicited that the use of traditional cold steel dissection and ties method had significantly decreased for both consultants (*r* = −0.958, *p* < 0.001) and specialist registrars (*r* = −0.817, *p* = 0.004) within the study period. In contrast, the use of bipolar diathermy (for dissection and haemostasis) had significantly increased for both consultants (*r* = 0.886, *p* = 0.001) and specialist registrars (*r* = 0.665, *p* = 0.036). Tables 2 and 3 summarise the trends of tonsillectomy techniques utilised between 2003 and 2012 in Wales for consultants and specialist registrars, respectively. Figures 2 and 3 depict the trends in tonsillectomy techniques utilised by consultants and specialist trainees, respectively, in Wales between 2003 and 2012.

Tonsil surgery has evolved greatly. Interestingly, its earliest report in the first century AD was by Aulus Cornelius Celsus, who first described removing the tonsil using a finger and washing the wound with vinegar or juice of comfrey [18]. “Hot” techniques for tonsillectomy first came into existence when Remington-Hobbs, in 1968, described the use of diathermy for tonsil dissection and haemostasis in a report of more than 5000 cases [19]. Coblation was later introduced in 2001, and a plethora of other techniques such as the harmonic scalpel, argon beam coagulator, and laser ablation have also been developed, each with their own risks and benefits.

The choice of technique to employ is inherently based on a number of factors, including risk of intraoperative blood loss, posttonsillectomy infection and haemorrhage, operating time, surgeons experience and preference, severity of postoperative pain, and early return to normal activities. A reduction in these parameters is thought to improve patient recovery time and satisfaction as well as bearing obvious social and economic implications [7].

The UK National Prospective Tonsillectomy Audit (NPTA) [9] included 33,921 patients. It reported varying primary (within the first 24 hours) haemorrhage rates from 0.4% (bipolar diathermy) to 1.1% (monopolar diathermy). Secondary (beyond 24 hours postoperatively) haemorrhage rates ranged from 1% (cold steel and ties) to 5.5% (monopolar diathermy). There was, however, no strong statistical evidence for differences in return to theatre (RTT) rates between most techniques. Only coblation had an elevated risk that was statistically significant with an adjusted odds ratio of 2.84 (95% CI 1.56–5.17) when compared to cold steel and ties. This is in contrast to findings from a recent study by Söderman et al. [20], which included 15,734 patients. They reported an early haemorrhage rate of 3% for cold steel dissection and ties and a comparable 3.9% for coblation tonsillectomy. Late bleeding rates were 3.3% and 9.9% for cold steel with ties and coblation, respectively. Söderman et al. found the adjusted odds ratio for secondary RTT for patients undergoing coblation tonsillectomy was 2.20 (95% CI 0.93–5.19). The authors concluded that the risk for RTT was higher for all hot techniques except for coblation. Tomkinson et al. [21] found that methods utilising heat resulted in a significantly greater adjusted odds of secondary RTT as compared with cold steel dissection. This value ranged from 2.7 times greater for cold steel dissection that used diathermy and ties for haemostasis to a 7.0-fold increased likelihood of secondary RTT associated with coblation.

The NPTA recommended that hot techniques should be used with caution especially when used as a dissection tool and that trainee surgeons should become competent at cold steel dissection and cold haemostasis prior to embarking on other techniques in tonsillectomy. Being an observational study, the results of the NPTA are susceptible to bias. Inclusion within the study was incomplete, and this is apparent on comparison with Hospital Episode Statistics data. Lowe et al. [22] suggest one cause for this discrepancy as being the need for informed consent. The incomplete inclusion may have underestimated the differences in haemorrhage rates. Secondly, the results of the NPTA may be skewed by the modified definition of primary and secondary haemorrhage. A haemorrhage occurring after the first 24 hours postoperatively but during the initial stay is included in the NPTA's definition of primary haemorrhage but would be considered a secondary haemorrhage in other studies. This would result in minor bleeds not requiring intervention, unlikely to be recorded in the NPTA database. The difference in definition of postoperative haemorrhage also makes direct comparison of haemorrhage rates between the NPTA and other studies difficult.

Despite the national recommendations from the controversial, yet informative, NPTA, our prospective observational study of over 19,000 tonsillectomies within a national cohort elicits a sustained increase in the use of bipolar diathermy and coblation techniques for tonsillectomy with a simultaneous fall in the utilisation of the gold standard cold steel dissection and ties. The underlying reasons for the observation of increased use of hot tonsillectomy techniques ascertained by the present study may include shorter procedural time and less intraoperative blood loss [23] as such techniques allow virtually simultaneous dissection and haemostasis. This is a particularly important consideration in young children where minimising blood loss is paramount. The NPTA suggests another reason for the preference of bipolar dissection technique amongst trainee surgeons is that the technique is quicker to learn and requires less dexterity than cold steel dissection with the ligation of vessels [9].

The postulation of less intraoperative blood loss with hot tonsillectomy techniques is supported by evidence from a clinical trial undertaken by Kousha et al. [24], which demonstrated significantly less intraoperative blood loss and shorter operative duration with bipolar diathermy tonsillectomy compared to cold steel dissection with ties. In another prospective study, Pang [25] also made the observation of significantly shorter operating time and lower intraoperative blood loss using the bipolar diathermy technique compared to cold steel dissection and ties. Similarly, in a randomized controlled trial, Di Rienzo Businco and Coen Tirelli [26] ascertained that coblation tonsillectomy resulted in less intraoperative blood loss than cold steel dissection and ties.

Conversely, Hilton [11] argues that the perceived saving of operating time between diathermy dissection and cold steel with ties is no more than a few minutes, thereby unlikely to alter the potential throughput of cases on a standard operating list. A Cochrane review [23] addressed the issue of intraoperative haemorrhage, identifying a mean difference of about 21 mL per patient when comparing cold dissection with diathermy dissection (less bleeding). This figure is only 2% of the circulating blood volume of an average 2-year-old child, and most tonsillectomies are done on older children and adults.

Nevertheless, it may be argued that hot tonsillectomy techniques confer benefits in terms of reduced postoperative pain and earlier return to normal diet and hence the increase in their uptake. In a prospective randomized controlled trial (RCT) of 92 adults undergoing tonsillectomy for recurrent tonsillitis allocated to either coblation or cold steel dissection, Philpott et al. demonstrated there was no significant difference between the two groups in terms of postoperative pain and notably found the cold steel dissection group returned earlier to normal eating [7]. Conversely, in a prospective RCT of 19 adult patients undergoing a coblation tonsillectomy on one side and cold steel dissection on the other, postoperative pain levels in coblation tonsillectomy were significantly lower for the first 3 days following which the difference failed to show statistical significance [27]. Similarly, a prospective randomised double blinded trial including 79 paediatric patients aged between 4 and 16 years found no significant difference for pain scores, at the various time points measured in either the coblation or cold steel dissection group except for the 6th day in favour of the coblation technique [28]. In this study, the coblation group also required less analgesia in the first 12 hours postoperatively. In similarity, a prospective single blinded controlled trial including 34 patients found postoperative pain scores significantly less at 6 hours after operation in the coblation tonsillectomy group as compared to cold steel tonsillectomy. However, there were no differences in the pain scores on days 1, 2, and 3 postoperatively [29].

A retrospective audit of coblation tonsillectomies performed by a single surgeon over a 10-year period in Australia reported a 3.4% readmission rate with secondary haemorrhage and 1.3% RTT rate (0.4% primary haemorrhage and 0.9% secondary haemorrhage). The secondary haemorrhage rate and RTT are comparable to the NPTA; however, the primary RTT is vastly improved at 0.4% compared to 1.1% as seen in the NPTA. The authors conclude that coblation can be a safe method for tonsillectomy with low complication rates when performed by an experienced surgeon [30]. Moreover, in a meta-analysis of 24 prospective randomised controlled studies by Mösges et al. [31] comprising data of 796 patients who had undergone coblation tonsillectomy, the overall haemorrhage rate was 4.1% with a 95% confidence interval from 2.8 to 5.5%. This highlights that coblation is relatively safe and effective with bleeding rates that are comparable to other techniques. That noted, a Cochrane review reported inadequate evidence due to poor quality trials to determine whether coblation tonsillectomy is better or worse than other tonsillectomy techniques [32].

As is true for other surgical techniques, adequate training is required for surgeons new to coblation. Carney et al. [33] studied 2062 tonsillectomies and concluded the presence of a “learning curve” in that surgeon experience was statistically significant and accounted for 60% of the variability in primary bleeding rate and 8% of the variability in secondary bleeding rate. The NPTA recommended training prior to embarking on “hot” techniques and it may be a possibility that data presented in the current study reflects that very situation is occurring in Wales.

The present study is of course not without limitations. Being an observational study, the results of the current study are susceptible to bias; firstly, the study excluded a substantial number of patients with incomplete records. This could have undermined the differences in operative techniques used if the excluded patients underwent a technique to minimise various risks such as postoperative pain or intraoperative blood loss. Ultimately the technique employed is the surgeons' choice and may be based on patient or treatment characteristics as well as a personal preference. Moreover, although this study examined trends in how relatively new tonsillectomy techniques have been adopted using longitudinal data, it does not examine how these techniques and devices affect patient outcomes after tonsillectomy, and most importantly it does not elucidate if there had been any concomitant trends in posttonsillectomy haemorrhage rates. This will require further detailed study.

The findings of this study are in some ways comparable to published data from the National Tonsil Surgery Register in Sweden. In a prospective observational study analysing some 15,734 tonsillectomies between 2009 and 2013, cold steel dissection with diathermy haemostasis was the dominating technique and used in about two-thirds of the patients [20]. Bipolar diathermy was used in 15.7%, and coblation was the third most frequent technique (9.1%). Cold steel with cold haemostasis was only used in 7.4% during the study period. Interestingly, this study ascertained early and late postoperative haemorrhage rates for these techniques and demonstrated unambiguously that cold steel dissection and cold haemostasis should be considered the gold standard technique for tonsillectomy in terms of having the lowest risk of late postoperative haemorrhage, in spite of falling trends in its use.

## 4. Conclusions

Despite data from several studies reporting an increase in the likelihood of secondary haemorrhage with hot tonsillectomy techniques, there remains an increasing trend of hot tonsillectomy techniques being utilised in Wales. Inevitably, the decision of which technique to use is based on surgeon and patient factors and may be best decided on a case by case basis. However, as with all aspects of modern surgery, this decision should be evidence-based. The evidence remains insufficient but currently does not support the increased use of hot tonsillectomy techniques seen in this large cohort of patients. This trend and the underlying reasons for it should be examined further and reflected upon.

## Figures and Tables

**Table 1:** Trends in tonsillectomy techniques utilised in Wales between 2003 and 2012.

Tonsillectomy technique	% change	Pearson's correlation (*r*)	*p* value
Coblation	+120	0.825	0.003
Cold steel + ties	−60	−0.939	<0.001
Cold steel + both (diathermy haemostasis + ties)	+35	0.706	0.022
Bipolar diathermy (dissection & haemostasis)	+84	0.762	0.010
Laser	−115	−0.748	0.013
Ultrasonic	−133	−0.786	0.007
Monopolar diathermy	−103	−0.659	0.038

**Table 2:** Trends in tonsillectomy techniques utilised by consultants in Wales between 2003 and 2012.

Tonsillectomy technique	% change	Pearson's correlation (*r*)	*p* value
Coblation	+20	0.327	0.356
Cold steel + ties	−59	−0.958	<0.001
Cold steel + both (diathermy haemostasis + ties)	+22	0.704	0.023
Bipolar diathermy	+180	0.886	0.001

**Table 3:** Trends in tonsillectomy techniques utilised by specialist registrars in Wales between 2003 and 2012.

Tonsillectomy technique	% change	Pearson's correlation (*r*)	*p* value
Coblation	+2621	0.846	0.002
Cold steel + ties	−52	−0.817	0.004
Cold steel + both (diathermy haemostasis + ties)	+12	0.414	0.234
Bipolar diathermy	+252	0.665	0.036

## References

[1] Younis R. T., Lazar R. H. (2002). History and current practice of tonsillectomy. *Laryngoscope*.

[2] Koempel J. A. (2002). On the origin of tonsillectomy and the dissection method. *The Laryngoscope*.

[3] Charaklias N., Mamais C., Kumar B. N. (2011). The art of tonsillectomy: the UK experience for the past 100 years. *Otolaryngology—Head and Neck Surgery*.

[4] Ballantynem J. C., Harrison D. F. N. (1986). *Rob & Smith's Operative Surgery: Nose and Throat*.

[5] Roy A., De La Rosa C., Vecchio Y. A. (1976). Bleeding following tonsillectomy. A study of electrocoagulation and ligation techniques. *Archives of Otolaryngology*.

[6] Temple R. H., Timms M. S. (2001). Paediatric coblation tonsillectomy. *International Journal of Pediatric Otorhinolaryngology*.

[7] Philpott C. M., Wild D. C., Mehta D., Daniel M., Banerjee A. R. (2005). A double-blinded randomized controlled trial of coblation versus conventional dissection tonsillectomy on post-operative symptoms. *Clinical Otolaryngology*.

[8] Fox R., Temple M., Owens D., Short A., Tomkinson A. (2008). Does tonsillectomy lead to improved outcomes over and above the effect of time? A longitudinal study. *Journal of Laryngology and Otology*.

[9] British Association of Otorhinolaryngologists—Head and Neck Surgeons Comparative Audit Group and the Clinical Effectiveness Unit. The Royal College of Surgeons of England (2005). *National Prospective Tonsillectomy Audit Final Report*.

[10] Windfuhr J. P., Chen Y.-S. (2002). Incidence of post-tonsillectomy hemorrhage in children and adults: a study of 4,848 patients. *Ear, Nose & Throat Journal*.

[11] Hilton M. (2004). Tonsillectomy technique—tradition versus technology. *The Lancet*.

[12] Sarny S., Ossimitz G., Habermann W., Stammberger H. (2011). Hemorrhage following tonsil surgery: a multicenter prospective study. *The Laryngoscope*.

[13] O'Leary S., Vorrath J. (2005). Postoperative bleeding after diathermy and dissection tonsillectomy. *The Laryngoscope*.

[14] Blanchford H., Lowe D. (2013). Cold versus hot tonsillectomy: state of the art and recommendations. *ORL*.

[15] The Surgical Instrument Surveillance Programme Working Group (2006). Surgical instrument surveillance programme. *All Wales Annual Tonsillectomy Surveillance Report*.

[16] Patient Episode Database for Wales *Annual PEDW Data Tables*. http://www.infoandstats.wales.nhs.uk/page.cfm?orgid=869&pid=41010&subjectlist=Main%2bOperation%2b%284%2bcharacter%2bdetail%29&patientcoverlist=0&period=0&keyword=&action=Search.

[17] Office of Population Censuses and Surveys (1990). *Tabular List of the Classification of Surgical Operations and Procedures*.

[18] McGuire N. G. (1967). A method of guillotine tonsillectomy with an historical review. *The Journal of Laryngology and Otology*.

[19] Remington-Hobbs C. (1968). Diathermy in dissection tonsillectomy and retrograde dissection adenoidectomy. *The Journal of Laryngology and Otology*.

[20] Söderman A.-C. H., Odhagen E., Ericsson E. (2015). Post-tonsillectomy haemorrhage rates are related to technique for dissection and for haemostasis. An analysis of 15734 patients in the National Tonsil Surgery Register in Sweden. *Clinical Otolaryngology*.

[21] Tomkinson A., Harrison W., Owens D., Harris S., McClure V., Temple M. (2011). Risk factors for postoperative hemorrhage following tonsillectomy. *Laryngoscope*.

[22] Lowe D., van der Meulen J., Cromwell D. (2007). Key messages from the national prospective tonsillectomy audit. *The Laryngoscope*.

[23] Pinder D. K., Wilson H., Hilton M. P. (2011). Dissection versus diathermy for tonsillectomy. *Cochrane Database of Systematic Reviews*.

[24] Kousha A., Banan R., Fotoohi N., Banan R. (2007). Cold dissection versus bipolar electrocautery tonsillectomy. *Journal of Research in Medical Sciences*.

[25] Pang Y. T. (1995). Paediatric tonsillectomy: bipolar electrodissection and dissection/snare compared. *The Journal of Laryngology and Otology*.

[26] Di Rienzo Businco L., Coen Tirelli G. (2008). Paediatric tonsillectomy: radiofrequency-based plasma dissection compared to cold dissection with sutures. *Acta Otorhinolaryngologica Italica*.

[27] Polites N., Joniau S., Wabnitz D. (2006). Postoperative pain following coblation tonsillectomy: randomized clinical trial. *ANZ Journal of Surgery*.

[28] Parker D., Howe L., Unsworth V., Hilliam R. (2009). A randomised controlled trial to compare postoperative pain in children undergoing tonsillectomy using cold steel dissection with bipolar haemostasis versus coblation technique. *Clinical Otolaryngology*.

[29] Izny Hafiz Z., Rosdan S., Mohd Khairi M. D. (2014). Coblation tonsillectomy versus dissection tonsillectomy: a comparison of intraoperative time, intraoperative blood loss and post-operative pain. *The Medical Journal of Malaysia*.

[30] Rogers M. A., Frauenfelder C., Woods C., Wee C., Carney A. S. (2015). Bleeding following coblation tonsillectomy: a 10-year, single-surgeon audit and modified grading system. *The Journal of Laryngology and Otology*.

[31] Mösges R., Hellmich M., Allekotte S., Albrecht K., Böhm M. (2011). Hemorrhage rate after coblation tonsillectomy: a meta-analysis of published trials. *European Archives of Oto-Rhino-Laryngology*.

[32] Burton M. J., Doree C. (2007). Coblation versus other surgical techniques for tonsillectomy. *The Cochrane Database of Systematic Reviews*.

[33] Carney A. S., Harris P. K., MacFarlane P. L., Nasser S., Esterman A. (2008). The coblation tonsillectomy learning curve. *Otolaryngology—Head and Neck Surgery*.

